# Association between the triglyceride–glucose index and severity of coronary artery disease

**DOI:** 10.1186/s12933-022-01606-5

**Published:** 2022-09-01

**Authors:** Xiang Wang, Wei Xu, Qirui Song, Zinan Zhao, Xuyang Meng, Chenxi Xia, Yibo Xie, Chenguang Yang, Pengfei Jin, Fang Wang

**Affiliations:** 1 Department of Cardiology, Institute of Geriatric Medicine, Beijing Hospital, National Center of Gerontology, Chinese Academy of Medical Sciences, Beijing, People’s Republic of China; 2 Graduate School of Peking Union Medical College, Chinese Academy of Medical Science, Beijing, China; 3 Emergency Center, State Key Laboratory of Cardiovascular Disease of China, National Center for Cardiovascular Diseases, Fuwai Hospital, National Clinical Research Center of Cardiovascular Diseases, Chinese Academy of Medical Sciences and Peking Union Medical College, Beijing, 100037 China; 4 Hypertension Center, State Key Laboratory of Cardiovascular Disease of China, National Center for Cardiovascular Diseases of China, Fuwai Hospital, Chinese Academy of Medical Sciences and Peking Union Medical College, Beijing, 100037 China; 5 Department of Pharmacy, Beijing Hospital, National Center of Gerontology; Institute of Geriatric Medicine, Chinese Academy of Medical Sciences; Beijing Key Laboratory of Assessment of Clinical Drugs Risk and Individual Application (Beijing Hospital), Beijing, People’s Republic of China; 6 Department of Information Center, Institute of Geriatric Medicine, Beijing Hospital, National Center of Gerontology, Chinese Academy of Medical Sciences, Beijing, People’s Republic of China

**Keywords:** Triglyceride–glucose index, Diabetes mellitus, Coronary artery disease

## Abstract

**Background:**

The triglyceride–glucose (TyG) index, which is a reliable surrogate marker of insulin resistance (IR), has been associated with cardiovascular diseases. However, evidence of the impact of the TyG index on the severity of coronary artery disease (CAD) is limited. This study investigated the relationship between the TyG index and CAD severity of individuals with different glucose metabolic statuses.

**Methods:**

This study enrolled 2792 participants with CAD in China between January 1, 2018 and December 31, 2021. All participants were divided into groups according to the tertiles of the TyG index as follows: T1 group, TyG index < 6.87; T2 group, TyG index ≥ 6.87 to < 7.38; and T3 group, TyG index ≥ 7.38. The glucose metabolic status was classified as normal glucose regulation, pre-diabetes mellitus (pre-DM), and diabetes mellitus according to the standards of the American Diabetes Association. CAD severity was determined by the number of stenotic vessels (single-vessel CAD versus multi-vessel CAD).

**Results:**

We observed a significant relationship between the TyG index and incidence of multi-vessel CAD. After adjusting for sex, age, body mass index, smoking habits, alcohol consumption, hypertension, estimated glomerular filtration rate, antiplatelet drug use, antilipidemic drug use, and antihypertensive drug use in the logistic regression model, the TyG index was still an independent risk factor for multi-vessel CAD. Additionally, the highest tertile of the TyG group (T3 group) was correlated with a 1.496-fold risk of multi-vessel CAD compared with the lowest tertile of the TyG group (T1 group) (odds ratio [OR], 1.496; 95% confidence interval [CI], 1.183–1.893; P < 0.001) in the multivariable logistic regression model. Furthermore, a dose–response relationship was observed between the TyG index and CAD severity (non-linear P = 0.314). In the subgroup analysis of different glucose metabolic statuses, the T3 group (OR, 1.541; 95% CI 1.013–2.344; P = 0.043) were associated with a significantly higher risk of multi-vessel CAD in individuals with pre-DM.

**Conclusions:**

An increased TyG index was associated with a higher risk of multi-vessel CAD. Our study indicated that TyG as an estimation index for evaluating IR could be a valuable predictor of CAD severity, especially for individuals with pre-DM.

## Background

Coronary artery disease (CAD), a cardiovascular disease (CVD) caused by coronary artery stenosis, is the main cause of death globally [1]. Because of aging and unhealthy lifestyle habits, morbidity and mortality caused by CAD are increasing; furthermore, CAD leads to a serious public health burden [2].

Recently, the incidences of type 2 diabetes mellitus (T2DM) and insulin resistance (IR) have increased remarkably with the improvement of living standards [3–5]. T2DM is a known risk factor that affects CAD progression and treatment strategies [6–8]. IR is a critical mechanism in the development of diabetes mellitus (DM) and has been broadly considered as a risk factor for atherosclerotic cardiovascular diseases [9–11]. The triglyceride–glucose (TyG) index, which is calculated as follows: Ln [fasting triglycerides (mg/dL) × fasting plasma glucose (mg/dL) / 2], is considered a dependable surrogate marker of IR [12, 13]. Previous studies have shown a significant relationship between the TyG index and incidence of CVDs, including coronary artery stenosis, coronary artery calcification, and carotid artery atherosclerosis, despite the presence of diabetes [14–17]. A recent large-scale study performed in Chinas suggested that an increased TyG index is independently correlated with a higher risk of myocardial infarction (MI) and emphasized the importance of monitoring the TyG index to distinguish individuals at high risk for MI [18].

Coronary angiography (CAG) as the gold standard for diagnosing CAD is an accurate and widely used imaging modality that aims to identify the number and degree of coronary artery stenosis. Participants with ≥ 50% lumen stenosis in at least one major coronary artery based on CAG findings were diagnosed with CAD [19]. Furthermore, the severity of CAD was based on the number of stenotic vessels and has a crucial role in the prognosis of CVD. Participants with multi-vessel CAD are at higher risk for CVD than those with single-vessel CAD, especially those with an abnormal glucose metabolic status [19, 20]. A recent study suggested that the TyG index is associated with the risk of multi-vessel CAD for the DM population, but not with that for individuals with pre-diabetes mellitus (pre-DM) or normoglycemia (NGR) [19]. However, the results might be attributable to a lack of power because of the small population enrolled in that study. Evidence of the effect of the TyG index on CAD severity is limited. Therefore, this study aimed to explore the relationship between the TyG index and CAD severity in a large cohort of participants with CAD and different glucose metabolic statuses.

## Methods

### Ethics statements

This retrospective observational cohort study conformed to the Declaration of Helsinki and was approved by the Ethics Committee of Beijing Hospital. Written informed consents were obtained from all participants.

### Study design and population

**Fig. 1:**
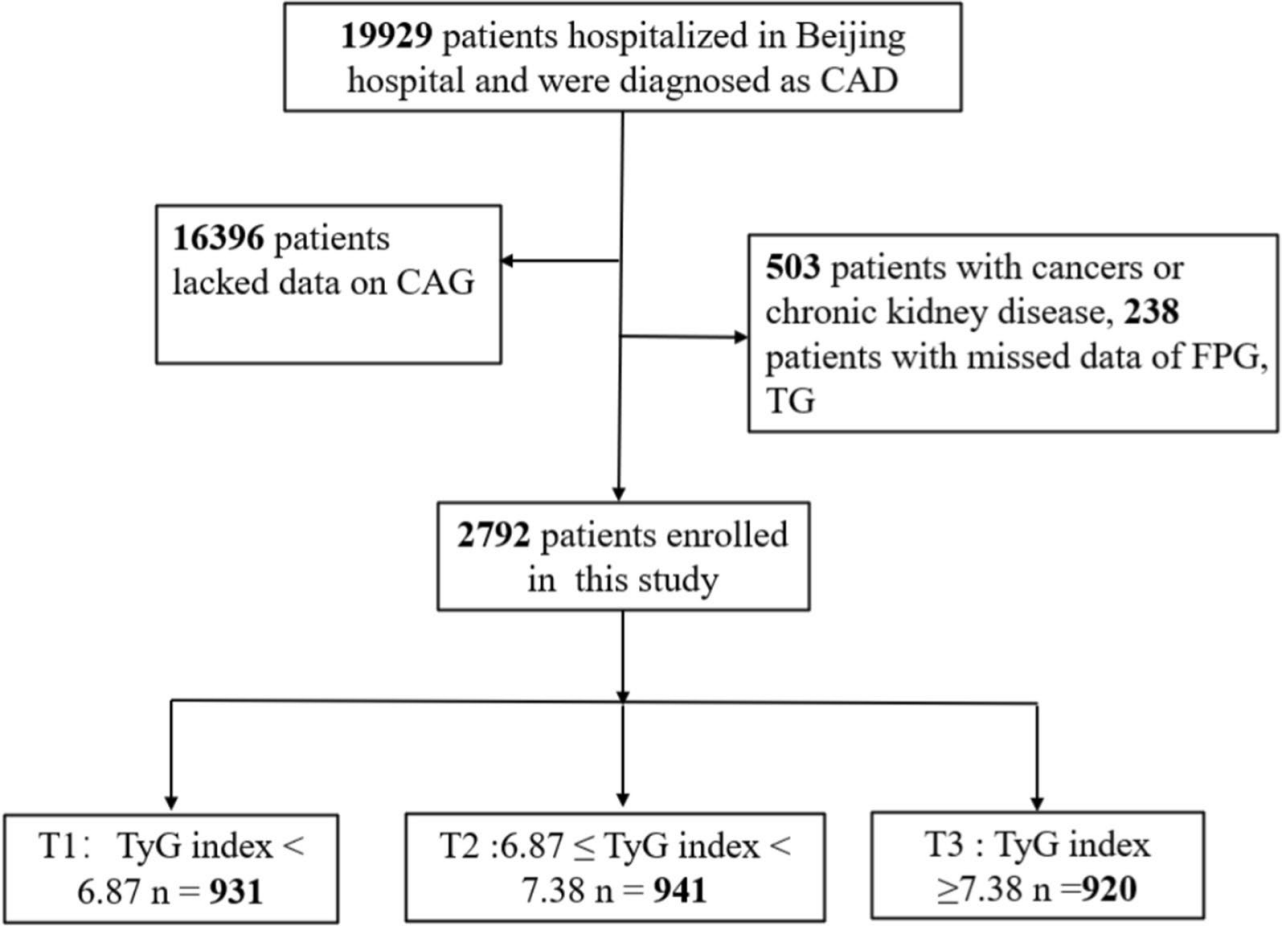
Flowchart of study participants. CAD, coronary artery disease; CAG, coronary angiography; FPG, fasting plasma glucose; TyG, triglyceride–glucose

We enrolled 19,929 participants hospitalized at Beijing Hospital who were diagnosed with CAD from January 1, 2016 to December 30, 2021. We excluded 16,396 participants who lacked data regarding CAG, 503 participants with cancer or chronic kidney disease, and 238 participants with missing fasting plasma glucose (FPG) and triglyceride data (Fig. 1). Finally, 2792 participants were included in the final statistical analysis that investigated the relationship between the TyG index and CAD severity. We divided the enrolled participants into three groups according to the tertiles of the TyG index as follows: T1 group, TyG index < 6.87 (n = 931); T2 group, TyG index ≥ 6.87 to < 7.38 (n = 941); and T3 group, TyG index ≥ 7.38 (n = 920).

### Measurements and definitions

All sociodemographic characteristics, medical history, medical imaging data, and blood sample analysis results of the participants were collected from the medical records. Sociodemographic characteristics included age, sex, height, weight, smoking status, and drinking. The medical history included a history of chronic kidney disease, cancer, diabetes, and hypertension. Antihypertensive, antiplatelet, and antilipidemic medications were recorded. Blood samples from all participants were collected after at least 8 h of fasting. Laboratory parameters, including FPG, creatinine, total cholesterol (TC), high-density lipoprotein cholesterol (HDL-C), and low-density lipoprotein cholesterol (LDL-C), were measured using the LABOSPECT 008 system (Hitachi, Tokyo, Japan), and the glycated hemoglobin A1c (HbA1c) level was determined using high-performance liquid chromatography (G8; TOSOH, Tokyo, Japan) in the laboratory of Beijing Hospital. The body mass index (BMI) was calculated as weight (kg) divided by the squared height (m^2^), and the estimated glomerular filtration rate (eGFR) was calculated according to the Chronic Kidney Disease Epidemiology Collaboration creatinine equation [21]. CAG was performed by experts who were blinded to the study protocol before an evaluation using percutaneous femoral arteriography.

The TyG index was calculated using the following equation: Ln [TG (mg/dL) × FPG (mg/dL) / 2].

CAD was referred to as at least one major coronary artery with ≥ 50% stenosis evaluated by CAG, including the left anterior descending, left circumflex, and right coronary arteries. The number of coronary arteries with ≥ 50% stenosis indicated the CAD severity [19]. Participants with one major coronary artery with ≥ 50% stenosis were defined as having single-vessel CAD, whereas multi-vessel CAD was considered when participants had more than two coronary arteries with ≥ 50% stenosis. According to the American Diabetes Association criteria, diabetes was diagnosed when participants had the following: an FPG level ≥ 7.0 mmol/L; 2-h plasma glucose level ≥ 11.1 mmol/L according to the oral glucose tolerance test; HbA1c ≥ 6.5%; or diabetes history. Pre-DM was diagnosed when participants without self-reported DM had an FPG level ranging from 5.6 to 6.9 mmol/L, 2-h plasma glucose level ranging from 7.8 to 11.0 mmol/L, or HbA1c level ranging from 5.7% to 6.4%. NGR was considered when participants did not have diabetes or pre-DM [22].

### Statistical analysis

Continuous variables are described as the mean ± standard deviation or median and interquartile range (25%–75%). Categorical variables are described as the number or percentage. The one-way analysis of variance or Kruskal–Wallis test was used to compare the baseline variables of the TyG index tertiles when appropriate, and the chi-square test was performed to compare the categorical variables among groups.

To analyze the association between the TyG index and CAD severity (single-vessel CAD versus multi-vessel CAD), odds ratios (ORs) and 95% confidence intervals (CIs) were calculated using a logistic regression analysis. Model 1 was unadjusted. Model 2 was adjusted for age and sex. Model 3 was adjusted for the variables in model 2 and further adjusted for antihypertensive medications, antidiabetic medications, FPG level, HDL-C level, LDL-level, triglyceride level, high-sensitivity C-reactive protein level, and eGFR. Moreover, restricted cubic splines were used to examine the shape of the associations between the baseline TyG index and CAD severity.

All statistical analyses were performed using SAS version 9.4 (SAS Institute, Inc., Cary, NC) and R version 4.0.3 (R Foundation for Statistical Computing). Statistical significance was set at P < 0.05.

## Results

### Baseline characteristics

The average age of the 2792 participants with CAD was 66 ± 10 years, and 69.02% were men. Table 1 shows the baseline characteristics based on tertiles of the TyG index. Compared with the other two groups, the T3 group participants tended to be younger and male and had higher BMI, FPG, LDL-C, TC, and eGFR values (all P < 0.05). Moreover, participants in the T3 group tended to have a history of hypertension and smoking (both P < 0.05). The proportions of DM, use of antihypertensive drugs, and multi-vessel CAD were higher in the T3 group than in the other groups (all P < 0.05).

**Table 1:** Baseline characteristics according to tertiles of the TyG index

	Total (n = 2792)	T1 (n = 931)	T2 (n = 941)	T3 (n = 920)	P-value
Age (years)	66 ± 10	68 ± 10	66 ± 10	63 ± 11	< 0.01
Male (n, %)	1927 (69.02%)	650 (69.82%)	643 (68.33%)	634 (68.91%)	< 0.01
SBP (mmHg)	136 ± 18	135 ± 18	136 ± 18	136 ± 19	0.11
DBP (mmHg)	77 ± 12	76 ± 12	77 ± 12	77 ± 12	0.03
FPG (mmol/l)	6.36 ± 2.22	5.29 ± 0.93	6.01 ± 1.40	7.81 ± 2.94	< 0.01
BMI (Kg/m2)	25.71 ± 3.34	24.74 ± 3.23	25.94 ± 3.11	26.45 ± 3.43	< 0.01
HDL-C (mg/dL)	1.04 ± 0.25	1.15 ± 0.27	1.03 ± 0.23	0.94 ± 0.21	< 0.01
LDL-C (mg/dL)	2.12 ± 0.80	1.89 ± 0.71	2.12 ± 0.74	2.36 ± 0.88	< 0.01
TC (mg/dL)	3.72 ± 0.93	3.45 ± 0.80	3.65 ± 0.84	4.07 ± 1.03	< 0.01
HbA1c (%)	6.59 ± 1.23	6.17 ± 0.87	6.45 ± 1.06	7.16 ± 1.45	< 0.01
eGFR (ml/min)	85.97 ± 17.08	86.24 ± 15.31	85.03 ± 16.33	86.65 ± 19.34	0.12
Smoking (n, %)	1299 (46.53%)	406 (43.61%)	432 (45.91%)	461 (50.11%)	0.02
Drinking (n, %)	1771 (63.43%)	584 (62.73%)	588 (62.49%)	599 (65.11%)	0.43
Hypertension (n, %)	1949 (69.81%)	595 (63.91%)	674 (71.63%)	680 (73.91%)	< 0.01
Glucose metabolism state					
NGR (n, %)	530 (18.98%)	275 (29.54%)	175 (18.60%)	80 (8.70%)	< 0.01
Pre-DM (n, %)	1038 (37.17%)	394 (42.32%)	399 (42.40%)	245 (26.63%)	< 0.01
DM (n, %)	1224 (43.84%)	262 (28.14%)	367 (39.00%)	595 (64.67%)	< 0.01
Medications					
Antiplatelets (n, %)	2643 (94.66%)	883 (94.84%)	883 (93.84%)	877 (95.33%)	0.34
Antihypertensive drugs (n, %)	2317 (82.99%)	728 (78.20%)	795 (84.48%)	794 (86.30%)	< 0.01
Antilipidemic drugs (n, %)	2629 (94.16%)	877 (94.20%)	878 (93.30%)	874 (95.0%)	0.30
Multi-vessel CAD (n, %)	2159 (77.33%)	688 (73.90%)	736 (78.21%)	735 (79.89%)	< 0.01

*TyG* triglyceride–glucose, *SBP* systolic blood pressure, *DBP* diastolic blood pressure, *FPG* fasting plasma glucose, *BMI* body mass index, *HDL-C* high-density lipoprotein cholesterol, *LDL-C* low-density lipoprotein cholesterol, *TC* total cholesterol, *HbA1c* glycated hemoglobin A1c, *eGFR* estimated glomerular filtration rate, *NGR* normoglycemia, *Pre-DM* pre-diabetes mellitus, *DM* diabetes mellitus, *CAD* coronary artery disease

Table 2 presents the baseline characteristics of those with single-vessel and those with multi-vessel CAD. A total of 2159 participants were diagnosed with multi-vessel CAD and evaluated using percutaneous CAG. Compared with participants with single-vessel CAD, those with multi-vessel CAD tended to be older and men. However, the eGFR and BMI values of participants with multi-vessel CAD were lower (both P < 0.05). Regarding the glucose metabolism statuses, the occurrence rate of multi-vessel CAD were 17.28%, 36.17%, and 46.55% for those with NGR, pre-DM, and DM, respectively. Moreover, the proportion of—antihypertensive drug use was higher for participants with multi-vessel CAD than for those with single-vessel CAD (both P < 0.05).

**Table 2:** Baseline characteristics according to single-vessel or multi-vessel CAD

	Total (n = 2792)	Single-vessel CAD (n = 633)	Multi-vessel CAD (n = 2159)	P-value
Age (years)	66 ± 10	65 ± 10	66 ± 10	< 0.01
Male (n,%)	1927 (69.02%)	359 (56.71%)	1568 (72.62%)	< 0.01
SBP (mmHg)	136 ± 18	136 ± 18	135 ± 18	0.58
DBP (mmHg)	77 ± 12	77 ± 11	77 ± 12	0.56
FPG (mmol/l)	6.36 ± 2.22	5.92 ± 1.68	6.49 ± 2.34	< 0.01
BMI (Kg/m2)	25.71 ± 3.34	25.79 ± 3.38	25.68 ± 3.32	< 0.01
HDL-C (mg/dL)	1.04 ± 0.25	1.10 ± 0.27	1.03 ± 0.25	0.92
LDL-C (mg/dL)	2.12 ± 0.80	2.12 ± 0.78	2.12 ± 0.81	0.92
TC (mg/dL)	3.72 ± 0.93	3.77 ± 0.90	3.71 ± 0.94	0.17
HbA1c (%)	6.59 ± 1.23	6.33 ± 1.03	6.67 ± 1.27	< 0.01
eGFR	85.97 ± 17.08	88.88 ± 14.02	85.11 ± 17.79	< 0.01
Smoking (n, %)	1299 (46.53%)	250 (39.49%)	1049 (48.59%)	< 0.01
Drinking (n, %)	1771 (63.43%)	394 (62.24%)	1377 (63.78%)	0.48
Hypertension (n, %)	1949 (69.81%)	412 (65.09%)	1537 (71.19%)	< 0.01
Glucose metabolism state				
NGR (n, %)	530 (18.98%)	157 (24.80%)	373 (17.28%)	< 0.01
Pre-DM (n, %)	1038 (37.8%)	257 (40.60%)	781 (36.17%)	< 0.01
DM (n, %)	1224 (43.84%)	219 (34.60%)	1005 (46.55%)	< 0.01
Medications				
Antiplatelets (n, %)	2643 (94.66%)	595 (94.00%)	2048 (94.86%)	0.40
Antihypertensive drugs (n, %)	2317 (82.99%)	491 (77.57%)	1826 (84.58%)	< 0.01
Antilipidemic drugs (n, %)	2629 (94.16%)	590 (93.21%)	2039 (94.44%)	0.25

*SBP* systolic blood pressure, *DBP* diastolic blood pressure, *FPG* fasting plasma glucose, *BMI* body mass index, *HDL-C* high-density lipoprotein cholesterol, *LDL-C* low-density lipoprotein cholesterol, *TC* total cholesterol, *HbA1c* glycated hemoglobin A1c, *eGFR* estimated glomerular filtration rate, *NGR* normoglycemia, *Pre-DM* pre-diabetes mellitus, *DM* diabetes mellitus, *CAD* coronary artery disease

### Association between the TyG index and severity of CAD

Table 3 describes the results of the logistic regression analysis. The univariate logistic regression analysis indicated that the TyG index was not statistically correlated with multi-vessel CAD. However, the T2 group (OR, 1.268; 95% CI 1.025–1.569; P = 0.029) and T3 group (OR, 1.403; 95% CI 1.129–1.745; P = 0.002) were at higher risk for multi-vessel CAD. After adjusting for age and sex in model 2, the TyG index as a continuous variable was an independent predictor of multi-vessel CAD (OR, 1.398; 95% CI 1.197–1.633; P < 0.001). Using the T1 group as a reference, the multivariate logistic regression analysis indicated that the risk of multi-vessel CAD for the T2 and T3 groups was 1.330-fold higher (OR, 1.330; 95% CI 1.061–1.668; P = 0.013) and 1.578-fold higher (OR, 1.578; 95% CI 1.249–1.994; P < 0.001), respectively. After adjusting for sex, age, BMI, smoking, drinking, hypertension, eGFR, antiplatelet drug use, antihypertensive drug use, and antilipidemic drug use, we found that the TyG index as a continuous variable was still an independent hazard factor for multi-vessel CAD (OR, 1.355; 95% CI 1.154–1.591; P < 0.001). Compared with the T1 group, which was regarded as the reference, the T2 group had a 1.283-fold risk of multi-vessel CAD (OR, 1.283; 95% CI 1.024–1.607; P = 0.031 ) in the multivariate logistic regression model, whereas the T3 group had a 1.496-fold risk of multi-vessel CAD (OR, 1.496; 95% CI 1.183–1.893; P < 0.001).

**Table 3:** Associations between the TyG index and severity of CAD

	Model 1			Model 2			Model 3		
	OR	95% CI	P-value	OR	95% CI	P-value	OR	95% CI	P-value
TyG index	0.825	0.559–1.216	0.331	1.398	1.197–1.633	< 0.001	1.355	1.154–1.591	< 0.001
T1	Reference			Reference			Reference		
T2	1.268	1.025–1.569	0.029	1.330	1.061–1.668	0.013	1.283	1.024–1.607	0.031
T3	1.403	1.129–1.745	0.002	1.578	1.249–1.994	< 0.001	1.496	1.183–1.893	< 0.001

Model 1: unadjusted

Model 2: adjusted for age and sex

Model 3: adjusted for sex, age, BMI, smoking, drinking, hypertension, eGFR, antiplatelet drug use, antilipidemic drug use, and antihypertensive drug use

*TyG* triglyceride–glucose, *CAD* coronary artery disease, *OR* odds ratio, *CI* confidence interval, *BMI* body mass index, *eGFR* estimated glomerular filtration rate

**Fig. 2:**
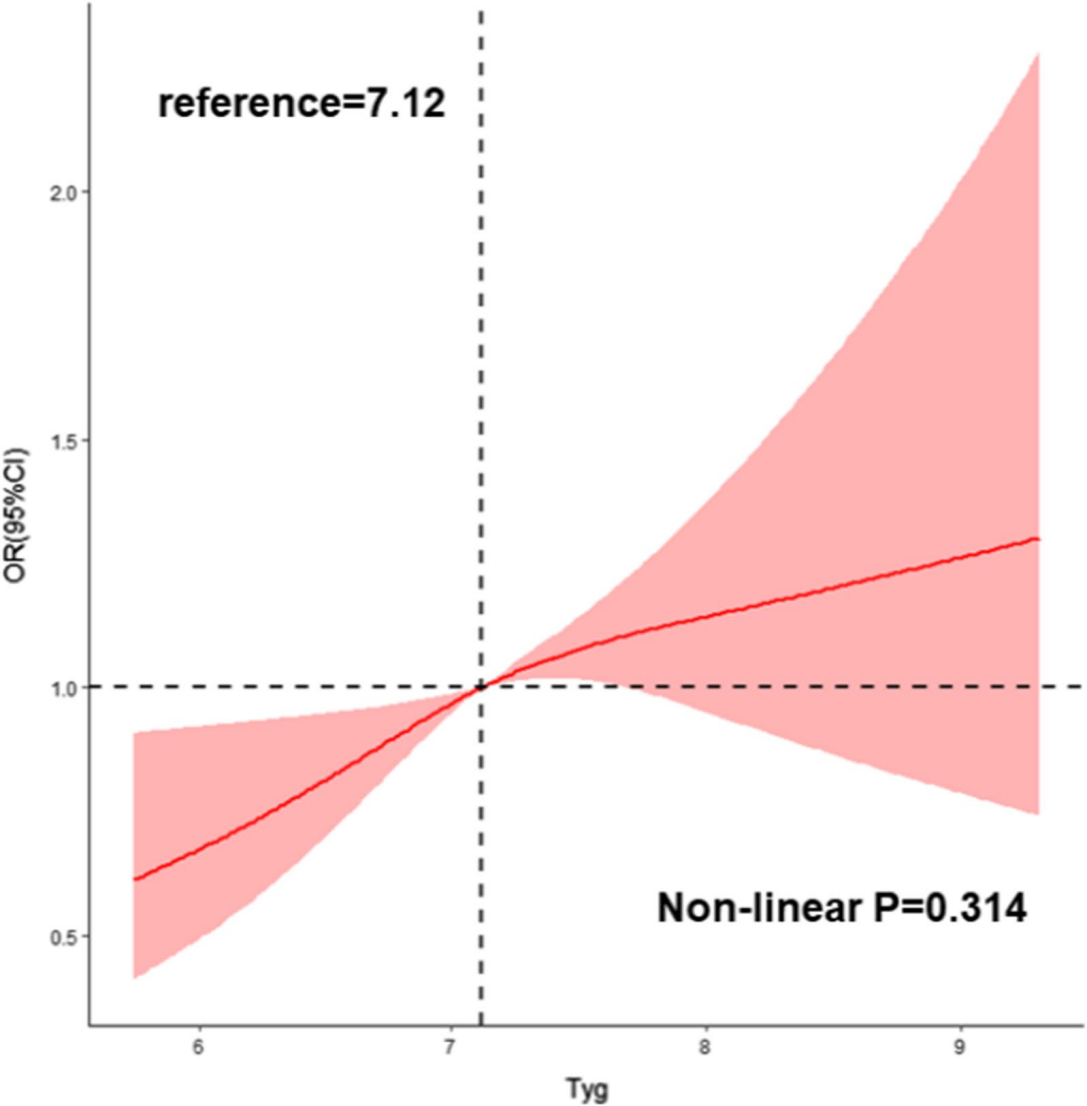
Restricted cubic splines for the odds ratio of multi-vessel CAD. CAD, coronary artery disease; OR, odds ratio; CI, confidence interval

The results of the restricted cubic splines are presented in Fig. 2. We observed a dose–response relationship between the TyG index and risk of multi-vessel CAD (non-linear P = 0.314).

Table 4 shows the relationship between the TyG index and CAD severity according to different diabetes statuses, including NGR, pre-DM, and DM. When adjusted for sex, age, BMI, smoking, drinking, hypertension, eGFR, antiplatelet drug use, antihypertensive drug use, and antilipidemic drug use in model 3, the TyG index as a continuous variable was an independent risk factor for multi-vessel CAD in subgroup of pre-DM (OR, 1.367; 95% CI 1.000–1.867; P = 0.049). In the pre-DM subgroup, the T3 group (OR, 1.541; 95% CI 1.013–2.344; P = 0.043) was associated with a significantly higher risk of multi-vessel CAD when the T1 group was used as the reference.

**Table 4:** Associations between the TyG index and severity of CAD according to different diabetes statuses

Glucose metabolism state	Model 1			Model 2			Model 3		
	OR	95% CI	P-value	OR	95% CI	P-value	OR	95% CI	P-value
NGR									
TyG index	0.825	0.559–1.216	0.331	0.929	0.617–1.398	0.723	0.846	0.551–1.299	0.444
T1	Reference			Reference			Reference		
T2	1.206	0.787–1.848	0.389	1.246	0.759–2.045	0.385	1.167	0.710–1.916	0.543
T3	0.660	0.393–1.109	0.117	0.739	0.400–1.366	0.335	0.684	0.371–1.262	0.225
Pre-DM									
TyG index	1.210	0.910–1.607	0.189	1.412	1.043–1.903	0.023	1.367	1.000–1.867	0.049
T1	Reference			Reference			Reference		
T2	1.161	0.844–1.597	0.359	1.230	0.859–1.761	0.258	1.217	0.860–1.724	0.268
T3	1.351	0.927–1.968	0.118	1.613	1.050–2.477	0.029	1.541	1.013–2.344	0.043
DM									
TyG index	1.162	0.931–1.449	0.185	1.230	0.982–1.542	0.072	1.214	0.963–1.530	0.100
T1	Reference			Reference			Reference		
T2	1.282	0.857–1.918	0.226	1.341	0.871–2.064	0.182	1.265	0.849–1.884	0.249
T3	1.300	0.901–1.875	0.161	1.390	0.934–2.069	0.104	1.321	0.921–1.914	0.141

Model 1: unadjusted

Model 2: adjusted for age and sex

Model 3: adjusted for sex, age, BMI, smoking, drinking, hypertension, eGFR, antiplatelet drug use, antilipidemic drug use, and antihypertensive drug use

*TyG* triglyceride–glucose, *CAD* coronary artery disease, *OR* odds ratio, *CI* confidence interval, *BMI* body mass index, *eGFR* estimated glomerular filtration rate, *NGR* normoglycemia, *Pre-DM* pre-diabetes mellitus, *DM* diabetes mellitus

## Discussion

In our study, a significant relationship between the TyG index and the occurrence of multi-vessel CAD, which represents the CAD severity, was observed. After adjusting for potential risk factors, including sex, age, BMI, smoking, alcohol consumption, hypertension, eGFR, antiplatelet drug use, antihypertensive drug use, and antilipidemic drug use, the TyG index was still an independent risk factor for multi-vessel CAD. Additionally, the highest tertile of the TyG group (T3 group) was correlated with a 1.496-fold risk of multi-vessel CAD compared with the lowest tertile of the TyG group (T1 group). To our best knowledge, the present study is the first to observe a dose–response relationship between the TyG index and risk of multi-vessel CAD. Furthermore, an increased TyG index was correlated with a significantly higher risk of multi-vessel CAD, especially for individuals with pre-DM.

Compared with single-vessel CAD, multi-vessel CAD is correlated with a higher risk of a worse prognosis even after percutaneous coronary intervention therapy [23]. Multi-vessel CAD increases the difficulty of percutaneous coronary intervention, reflects the severity of CAD, and has received constant attention in clinical practice. In our study, participants with multi-vessel CAD tended to be male and older, and they were more likely to have DM, hypertension, and a history of smoking. A retrospective cohort study showed that T2DM was independently related to a higher risk of multi-vessel CAD and severe CAD [20]. As a critical mechanism of DM, IR has a strong relationship with the development and progression of atherosclerotic cardiovascular diseases, especially CAD [10, 25]. IR indicates that insulin cannot function properly in target tissues, including the skeletal muscle, adipose tissue, and hepatic tissue [24]. Simental-Mendía et al. first suggested that the TyG index could be a surrogate for the Homeostatic Model Assessment to evaluate IR (HOMA-IR) [12]. Recently, the TyG index has been verified as a simple and dependable estimate index for IR and is comparable to the euglycemic-hyperinsulinemic clamp method, which is considered the gold standard for evaluating IR [26].

A large-scale retrospective study performed in Korea indicated that the group with the highest TyG index was at higher risk for stroke and MI [27]. Previous studies have demonstrated that the TyG index could be a useful marker of arterial stiffness and atherosclerosis [28, 29]. Moreover, a cohort study observed that the TyG index was related to the stenosis severity and number of stenosed coronary arteries [30]. Consistent with these studies, our study suggested that a higher TyG index was notably correlated with CAD severity in a relatively large cohort of participants with CAD. This finding indicated that the TyG index is expected to be a useful predictor of CAD severity before CAG is performed in clinical practice.

The relationship between IR and the risk of cardiovascular diseases in the established DM population has been controversial [31, 32]. Recently, a retrospective study suggested that the TyG index was correlated with the risk of multi-vessel CAD in the DM subgroup [19]. In contrast, our study did not observe a strong association between the TyG index and multi-vessel CAD in the DM subgroup. However, the definition of DM in the aforementioned study [19] excluded the self-reported DM history, which was different from our study. The results might have been influenced by the definitions and limited to their small study population. Consistent with our study, a previous study indicated that the TyG index is a useful marker for identifying IR in those without diabetes [33]. Another study that enrolled 5764 participants reported that IR parameters including the TyG index and HOMA-IR were not related to the risk of obstructive CAD in the DM population [31]. It is well-known that DM is characterized by hyperglycemia and IR. It is speculated that the probable mechanism of severe CAD may be more related to the glycemic status than IR in established diabetes [31]. The accuracy of this hypothesis should be confirmed by future studies. Our results support that intensive glycemia control is still the important strategy for preventing CAD in established diabetes [34].

Associations between pre-DM and cardiovascular diseases may differ according to the pre-DM criteria and ethnic variations [35]. A meta-analysis of 53 prospective studies demonstrated that pre-DM was correlated with an increased risk of composite CVD, including CAD [36]. In our study, the subgroup analysis suggested that a higher TyG index was significantly associated with an increased risk of multi-vessel CAD in the pre-DM group. According to previous studies, pre-DM was closely related to diffuse coronary stenosis compared to DM and NGR, which increases the complexity of the percutaneous coronary intervention and leads to a worse prognosis [37, 38]. Although pre-DM can be regarded as a risk factor for future CVD, it should be mentioned that not every participant needs drug therapy [36]. Therefore, it is vital to identify the phenotypes that require pharmacological intervention in the pre-DM population. The American Diabetes Association suggests considering drug therapy for participants with one or more risk factors, including high triglyceride concentration, HbA1c level > 42 mmol/mol, reduced HDL-C concentration, and hypertension [39]. Our results support the suggestions of the American Diabetes Association and demonstrate that TyG as a combined index of triglycerides and FPG for evaluating IR could be a useful marker for identifying participants at risk for severe CAD with pre-DM. CAD severity gradually increased with NGR, pre-DM, and DM in the present study. Regarding the results of the NGR subgroup, the routine assessments of the FPG, HbA1c, and TyG index are considered equally important to the early detection of pre-DM and IR.

## Strengths and limitations

This study included a relatively large cohort of participants with CAD. To the best of our knowledge, this is the first study to investigate the dose–response relationship of the TyG index and CAD severity of participants with CAD. However, this study also had several limitations. First, this study involved a single-center and only enrolled the Asian population; therefore, the results should be interpreted cautiously. Second, because of the inevitable inherent disadvantages of retrospective studies, we could not infer a causal relationship during this study; therefore, a prospective study is necessary to verify these findings in the future. Additionally, data regarding factors such as income, education, and the employment of participants were not collected during this study, which might have affected the results. Third, the medication durations and dosages of antiplatelet drugs, antilipidemic drugs, and antihypertensive drugs for this cohort were not collected, which might have resulted in bias for these factors in the logistic models.

## Conclusions

Our results demonstrated that an increased TyG index was correlated with a higher risk of multi-vessel CAD. A dose–response relationship was observed between the TyG index and risk of multi-vessel CAD. Our study indicated that TyG as an estimation index for evaluating IR could be a useful predictor of CAD severity, especially in the pre-DM population.

## Data Availability

The datasets generated and analysed during the current study are not publicly available due privacy and ethical restrictions but are available from the corresponding author on reasonable request.

## Abbreviations

CAD: Coronary artery disease
CVD: Cardiovascular disease
T2DM: Type 2 diabetes mellitus
IR: Insulin resistance
DM: Diabetes mellitus
ASCVD: Atherosclerotic cardiovascular diseases
TyG: Triglyceride–glucose
TG: Triglyceride
MI: Myocardial infarction
CAG: Coronary angiography
Pre-DM: Pre-diabetes mellitus
NGR: Normoglycemia
FPG: Fasting plasma glucose
TC: Total cholesterol
HDL-C: High-density lipoprotein cholesterol
HOMA-IR: Homeostatic Model Assessment to evaluate IR
LDL-C: Low-density lipoprotein cholesterol
HbA1c: Glycated hemoglobin A1c
BMI: Body mass index
eGFR: Estimated glomerular filtration rate
ADA: American Diabetes Association
OR: Odds ratio
CI: Confidence interval
PCI: Percutaneous coronary intervention
CHD: Coronary heart disease
